# Intelligence

**Published:** 1827-06

**Authors:** 


### INTELLIGENCE.

### MONTHLY REPORT OF PREVALENT DISEASES.

Ague, which we mentioned in our last report as more prevalent than usual, has continued to be frequently met with; and even common fever has assumed an aguish character.—We have examined an interesting case of a *blue boy*, nine months old, in whom a communication, large enough to admit the point of the forefinger, existed between the auricles and also between the ventricles, at the upper part of the interventricular septum.—The only other cases of interest which we have met with are two instances of Tic Douloureux, which are doing well under the use of Carbonate of Iron.

---

*On the Application of Leeches.* By S. G. Lawrance, Esq. Assistant Surgeon of the Royal Military Asylum, Chelsea.

Sir,—From a conviction that every endeavour to diminish human suffering, and to reconcile the mind to the remedies employed, by adapting them as much as possible to the feelings and prejudices of the patient, is an object well worthy of medical attention, I am induced to offer the following suggestions to your notice, and to request their insertion in your valuable and widely circulated Journal.

As in many diseases the local abstraction of blood by leeches is a very important means of cure, I am desirous of introducing to the notice of your readers a method which I have adopted and employed for some time, with much success, in procuring a larger quantity of blood from the application of a given number of leeches than is usually obtained.

The method consists in applying a cupping-glass, having a brass exhausting syringe fitted to it, over the bleeding orifices left by the leeches, immediately after they have fallen off, and exhausting the air, when the blood will flow freely into the glass, as in cupping with a scarificator. When the glass is nearly full, it is to be removed, cleansed, and reapplied, until it is perceived that the orifices cease to bleed.

By these means I have frequently been able to abstract, in a very short time, from three to four ounces of blood after the application of six or eight leeches to the temples, and from two to three ounces after the application of a similar number to the neck; but the quantity must necessarily vary according to the vascularity of the part to which the leeches are applied.

The size of the leeches also makes a considerable difference; for, in several experiments made for the express purpose, I found that I was able to abstract from one to two ounces of blood after the application of *two large leeches* to the temple, and only about half that quantity from *two small leeches* applied to the other temple of the same individual. I therefore recommend large leeches to be used.

Leeches also are often so scarce and so expensive, that it becomes a very desirable object to procure as much blood as possible from a small number of them.

The method above described appears to me to be peculiarly applicable to timid persons and to children, who always have a great dread of cutting instruments; likewise to females, who frequently express considerable aversion to cupping with the scarificator, in consequence of the permanent marks left by that instrument; whereas, the leech-bites soon heal, and after a few weeks the marks are scarcely discernible, and in most instances are totally obliterated.

It is also preferable to the customary mode of fomenting the bleeding orifices with warm water after the leeches have fallen off; for the continual oozing of blood which succeeds often keeps the patient in a very restless and uncomfortable state for many hours, and the quantity lost can never be correctly ascertained: whereas, after adopting the method proposed and above described, all bleeding from the orifices ceases, and the quantity of blood abstracted is accurately known.

In urgent or formidable cases, where it is absolutely necessary to take away twelve or fourteen ounces of blood, locally and speedily, cupping with the scarificator, in the usual way, must be resorted to; but in many others, such as inflammations of the eyes, slight affections of the head, &c. I am inclined to think the above method will be found of much utility. In the operation, however, it will be necessary to attend to the following directions:—

1st. To employ rather large-sized leeches, and so apply them that the orifices they leave may be sufficiently close to be included within the circumference of a small cupping-glass.

2dly. In exhausting the air, care must be taken not to do it too forcibly; for in that case the edge of the glass will act like a ligature, and prevent the free flow of blood. When the air is sufficiently exhausted, and the glass adheres, the syringe may be taken off, and a small brass cap screwed in its place over the valve, which will effectually prevent the ingress of air.

3dly. When the glass is removed with the blood, (which is to be emptied into a graduated glass measure,) it must be cleansed in hot water, and wiped before its reapplication; otherwise some fluid may be sucked up into the barrel of the syringe while exhausting the air, and thus obstruct the valve, and prevent its acting properly.

The instruments I have generally employed are those sold for cupping the temples, having only rather larger glasses.

I am, Sir, your obedient humble servant,

S. G. LAWRANCE.

*To the Editor of the*
*London Medical and Physical Journal.*

---

*Application of the Stomach Pump in a Case of Intoxication.*

Sir,—Having had an opportunity of trying the efficacy of the stomach pump, I send you the case as an additional instance of the important utility of this instrument.

John Albin, private soldier, second battalion Grenadier Guards, was brought into hospital on the evening of June 15th, 1826, by his comrades, in a state of total insensibility, from a public-house in the neighbourhood of the barracks, where he had been drinking whiskey to great excess. The symptoms were a livid and tumid countenance; stertorous breathing; a viscid, frothy discharge from the mouth; the pulse slow and laborious; extremities cold. Two pounds of blood were suddenly taken from the external jugular vein and arm at the same time. Half a drachm of the Sulphate of Zinc, dissolved in an ounce of water, was then administered by the mouth, and repeated in ten or fifteen minutes, without any material effect. The stomach pump, which had been sent for, was shortly after this introduced into the stomach, and about a pint of fluid extracted; which, from its strong smell, appeared to be nearly raw spirits. Some warm water being injected, was also extracted by the same means, still much impregnated with the smell of spirits. Within half an hour after this operation, the breathing became much more easy, and the pulse more free. He remained in a state of coma. Blisters were applied to the nape of the neck and exterior surface of the legs. Ten grains of Calomel in a bolus were directed to be given, and two ounces of Epsom Salts in solution, in divided doses, during the night.

At seven the following morning, he still remained in a comatose state. The pulse, however, had become more frequent, fuller, and softer; breathing easy; with a general warmth diffused over the surface of the body. He had passed his evacuations involuntarily. Towards noon he showed symptoms of returning sensation, attempted to speak, and made an effort to get up. Being placed on the night-chair, he passed a very copious evacuation; and from this period his recovery was progressive.

Your obedient servant, J. HARRISON.

*Westminster Hospital.*—Mr. Guthrie has been appointed fourth surgeon to this hospital. Mr. Harding, we understand, will not be opposed in his canvass for the office of assistant surgeon.

*St. George's Hospital.*—Sir E. Home has been appointed consulting surgeon, and Mr. Rose unanimously elected surgeon.

*Middlesex Hospital.*—Dr. Watson has been elected physician in the room of Dr. Southey.

*University of Glasgow.*—We understand that the medical chair is to be filled by Dr. Badham.

---

*Literary Notice.*—Dr. Rucco has in the press a work on the Science of the Pulse, as applied to the Practice of Medicine.

### METEOROLOGICAL JOURNAL,

*From April 20th, to May 20th,* 1827.

By Messrs. Harris and Co. Mathematical Instrument Makers, 50, High Holborn.

| April | Rain gauge. | Moon. | Thermom. | | | Barometer. | | De Luc's Hygrom. | | Winds. | | Atmospheric Variations. | | |
|---|---|---|---|---|---|---|---|---|---|---|---|---|---|---|
| | | | 9 A.M. | MAX. | MIN. | 9 A.M. | 10 P.M. | 9 A.M. | 10 P.M. | 9 A.M. | 10 P.M. | 9 *a.m.* | 2 *p.m.* | 10 *p.m.* |
| 20 | | | 44 | 50 | 44 | 29.53 | 29.47 | 96 | 90 | ENE | E | Cloudy | Cloudy | Cloudy |
| 21 | | | 45 | 49 | 41 | 29.39 | 29.49 | 98 | 98 | ENE | ENE | Rain | — | — |
| 22 | | | 43 | 45 | 37 | 29.56 | 29.66 | 88 | 82 | NE | NNE | Cloudy | — | — |
| 23 | | | 42 | 45 | 36 | 29.61 | 29.53 | 77 | .81 | N | S | — | — | Fair |
| 24 | | | 45 | 47 | 34 | 29.40 | 29.52 | 87 | 80 | SSW | SW | — | Sleet | — |
| 25 | | | 47 | 50 | 36 | 29.63 | 29.84 | 74 | 75 | SW v. | SW | Fair | Fair | — |
| 26 | | ● | 44 | 53 | 38 | 30.00 | 30.15 | 77 | 70 | W | SSW | — | — | — |
| 27 | | | 53 | 57 | 42 | 30.18 | 30.10 | 68 | 80 | SSE v. | E | — | Fine | — |
| 28 | | | 54 | 64 | 46 | 29.98 | 29.91 | 85 | 80 | E | E | — | — | — |
| 29 | | | 58 | 71 | 52 | 29.90 | 29.93 | 81 | 76 | E | E | — | — | — |
| 30 | | | 61 | 74 | 57 | 29.98 | 29.91 | 79 | 72 | WSW | W | — | — | — |
| May | | | | | | | | | | | | | | |
| 1 | | | 63 | 72 | 50 | 29.91 | 29.91 | 80 | 87 | W | S v. | — | — | Sl. Rain |
| 2 | | | 53 | 63 | 53 | 29.90 | 29.84 | 93 | 96 | ESE | ESE | Cloudy | — | Cloudy |
| 3 | | | 60 | 68 | 53 | 29.81 | 29.88 | 82 | 76 | W | W | Fair | — | Fair |
| 4 | | ☽ | 58 | 66 | 53 | 29 86 | 29.67 | 81 | 83 | WSW | SW | Cloudy | Cloudy | Cloudy |
| 5 | .65 | | 58 | 58 | 52 | 29.53 | 29.37 | 90 | 95 | SW | SSW | — | Rain | — |
| 6 | | | 53 | 56 | 44 | 29.21 | 29.34 | 95 | 92 | SSW | NNW | Rain | — | Rain |
| 7 | | | 49 | 55 | 37 | 29.55 | 29.57 | 72 | 80 | E | ENE | Cloudy | Cloudy | Fair |
| 8 | | | 46 | 50 | 39 | 29.72 | 29.76 | 78 | 75 | NE | E | — | — | Cloudy |
| 9 | | | 45 | 52 | 42 | 29.70 | 29.67 | 80 | 80 | ENE | ENE | — | — | — |
| 10 | | | 53 | 56 | 43 | 29.65 | 29.64 | 76 | 78 | ENE | ESE | — | — | Fair |
| 11 | | ○ | 52 | 59 | 42 | 29.66 | 29.83 | 78 | 81 | ENE | E | Fair | Fair | Cloudy |
| 12 | | | 50 | 57 | 40 | 29.96 | 29.97 | 75 | 81 | ENE | ENE | — | — | — |
| 13 | | | | 58 | 45 | 29.84 | 29.75 | 79 | 77 | NE | NNE | — | — | — |
| 14 | .10 | | 54 | 54 | 46 | 29.70 | 29.66 | 82 | 91 | NE | NW | — | Cloudy | Rain |
| 15 | | | 50 | 57 | 46 | 29.64 | 29.61 | 88 | 85 | WSW | SE | Cloudy | Fair | Cloudy |
| 16 | .17 | | 60 | 64 | 48 | 29.44 | 29.31 | 82 | 92 | E | SE | Fair | Cloudy | Rain |
| 17 | .35 | ☾ | 58 | 66 | 53 | 29.45 | 29.49 | 78 | 92 | SSE | E | — | Rain | — |
| 18 | | | 53 | 66 | 55 | 29.52 | 29.67 | 87 | 78 | SW | W | — | Fair | Fair |
| 19 | | | 65 | 67 | 55 | 29.80 | 29.90 | 75 | 71 | NW | W | — | Fine | — |

